# The Wistar Institute of Anatomy and Biology

**Published:** 1909-04

**Authors:** 


### CORRESPONDENCE

### The Wistar Institute of Anatomy and Biology

HUMAN EMBRYOS WANTED.

Philadelphia, Pa., March 2, 1909.

*Editor Buffalo Medical Journal:*

Sir—The Wistar Institute of Anatomy and Biology is making a strenuous effort to secure human embryological material. Last year we sent out a number of copies of the inclosed circular,[1] with the result that we obtained a number of perfect specimens of the younger stages so much desired for research work.

It is unnecessary to call your attention to the value of researches in a fundamental science like embryology, but I should like to call your attention to the fact that it is almost impossible for a research institution to obtain this material without the cooperation of men like yourself and I beg you, therefore, to give us such assistance in this matter as you may find possible.

We are prepared to send for specimens at any moment or to send neatly packed jars, with the necessary preserving fluid, to your office, hospital or to any place you may desire them.

We would like to secure specimens of all ages. The larger forms, from three months to term, may be sent by wrapping in a quantity of gauze and paper or by packing, without fluid, in a box if sent promptly so that they reach the laboratory within six hours, otherwise they should be placed in 10 per cent. formaldehyde. The smaller specimens should always be preserved in 10 per cent. formaldehyde.

Should you be inclined to assist us, will you not fill out the enclosed postal card and allow us to do what we can to save your time? Address all express or mail matter to The Wistar Institute of Anatomy, 36th street and Woodland avenue, Philadelphia, Pa.

The Wistar Institute, as you probably know, is an institution established for the promotion of researches in anatomy and allied subjects. Its museum is open freely to the public and its research laboratories are open without cost to any competent investigator.

M. J. Greenman, Director.

1. See next page.

TO PHYSICIANS AND SURGEONS.

The Wistar Institute of Anatomy and Biology appeals to you for coöperation in securing human embryos for scientific investigations.

No one, more than you, will appreciate the value of researches in anatomy and other fundamental sciences, for the advancement of your art. The Wistar Institute, therefore, begs you to assist in placing this material in the hands of embryologists who will make good use of it.

The collections of embryos and other specimens belonging to the Wistar Institute are always open to any competent investigator for inspection and study. Its museum serves as a depository for anatomical materials which may have served the purpose of one investigation and are to be stored for future study.

A moment's thought will convince you that in one month's time in a great city, more human embryological material than exists in any collection in the world, goes to waste. Will you help us to serve this material that American anatomists may study it?

HOW TO PRESERVE HUMAN EMBRYOS.

Place embryos, including membranes, immediately in a mixture of one part formalin and nine parts water. Never place an embryo in pure water; it damages it for histological purposes.

The Wistar Institute will send to your office, on request, a neatly boxed jar, with a quantity of formalin and full directions for preserving embryos. Operations for tubal or extra-uterine pregnancies are likely to yield the most perfect specimens.

Full credit will be given the collector in every case, and specimens will be reported upon promptly. The institute will gladly meet any expenses incurred.

If you have a specimen for the Institute, telephone Preston 2575 (Bell 'phone), and a messenger will call for it, or you may send it by express to

THE WISTAR INSTITUTE OF ANATOMY AND BIOLOGY,
36th Street and Woodland Avenue,
Philadelphia, Pa.

---

IS FOOD CONTAINING BENZOATES INJURIOUS TO HEALTH? E. E. Smith of New York analyzes Dr. H. W. Wiley's report of his "poison squad" experiments to determine the effect upon health of benzoaic acid and the benzoates, published in Bulletin No. 84 of the Bureau of Chemistry of the Department of Agriculture. He shows that the loss of weight which some of the men experimented upon underwent could be easily, and perhaps more logically, explained by other conditions than the finding of benzoic acid.—*Medical Record,* January 2, 1909.

